# The Roles of NO and H_2_S in Sperm Biology: Recent Advances and New Perspectives

**DOI:** 10.3390/ijms21062174

**Published:** 2020-03-21

**Authors:** Martin Kadlec, José Luis Ros-Santaella, Eliana Pintus

**Affiliations:** Department of Veterinary Sciences, Faculty of Agrobiology, Food and Natural Resources, Czech University of Life Sciences Prague, Kamýcká 129, 165 00 Praha 6-Suchdol, Czech Republic; kadlecmartin@af.czu.cz (M.K.); ros-santaella@ftz.czu.cz (J.L.R.-S.)

**Keywords:** gasotransmitters, hydrogen sulfide, interaction, metabolism, nitric oxide, spermatozoa

## Abstract

After being historically considered as noxious agents, nitric oxide (NO) and hydrogen sulfide (H_2_S) are now listed as gasotransmitters, gaseous molecules that play a key role in a variety of cellular functions. Both NO and H_2_S are endogenously produced, enzymatically or non-enzymatically, and interact with each other in a range of cells and tissues. In spite of the great advances achieved in recent decades in other biological systems, knowledge about H_2_S function and interactions with NO in sperm biology is in its infancy. Here, we aim to provide an update on the importance of these molecules in the physiology of the male gamete. Special emphasis is given to the most recent advances in the metabolism, mechanisms of action, and effects (both physiological and pathophysiological) of these gasotransmitters. This manuscript also illustrates the physiological implications of NO and H_2_S observed in other cell types, which might be important for sperm function. The relevance of these gasotransmitters to several signaling pathways within sperm cells highlights their potential use for the improvement and successful application of assisted reproductive technologies.

## 1. Introduction

Since the late 1980s, there has been increasing interest in the role of gaseous molecules in cellular physiology and pathology. Up until 1987, when Palmer et al. [1] identified the endothelium-derived relaxing factor to be nitric oxide (NO), this gas was regarded as a toxic agent. In the same year, Brüne and Ullrich [2] found that carbon monoxide (CO) inhibits platelet aggregation, enhancing guanylyl cyclase (GC) activity. With the discovery of the endogenous production of hydrogen sulfide (H_2_S) in rat and human brains [3], the term gasotransmitters emerged to set these three gases apart from the other known types of cellular messengers such as neurotransmitters and humoral factors [4]. Significant advances have been made in the area of gasotransmitters in the vascular [5,6], nervous [7,8], and digestive [9] systems. In contrast to the extensive literature available on NO [10,11], the role of H_2_S in male reproduction is less explored and deserves further attention [12]. This review aims to illustrate the role of NO and H_2_S in spermatozoa, and also includes recent advances in other cell types that may be potentially relevant to sperm biology. The spermatozoon represents one of the most diverse and specialized cells that originates from the spermatogonial cells in the seminiferous tubules of the testicles. Before leaving the male reproductive tract, the sperm cells undergo epididymal maturation, that is, a series of structural and biochemical changes resulting in the acquisition of fertilization ability and motility [13]. The full fertilization potential is not reached before the sperm cells go through the capacitation within the female reproductive tract. Capacitation involves plasma membrane changes initiated by the loss of cholesterol, also affecting the ion intracellular concentrations and the activity of specific enzymes (e.g., protein kinase A (PKA)). The series of these events results in different movement patterns, sperm hyperactivation, and finally allows the occurrence of an acrosomal reaction that is the exocytosis of specific enzymes from the sperm head covering vesicle, the acrosome [14]. 

### 1.1. NO Metabolism in Spermatozoa

The production of NO in cells is ensured by three isoforms of nitric oxide synthase (NOS) encoded by three different genes [15]. Irrespective of the NOS isoform, the substrates are L-arginine and oxygen (O_2_). The first NOS isoform is referred to as neuronal NOS (nNOS; NOS 1), as it was first discovered in neurons and its continuous expression is typical for peripheral and central neuronal cells. Through the action of nitric oxide, nNOS regulates the synaptic activity in the central nervous system and other functions, such as the regulation of blood pressure and smooth muscle relaxation. The second NOS isoform is referred to as inducible NOS (iNOS; NOS 2), since its expression may be induced by cytokines and lipopolysaccharides (LPS) [16]. The iNOS plays an important role in the immune system, as it generates a significant amount of NO, which helps to fight off pathological agents by the fragmentation of their DNA and the inhibition of iron-containing enzymes [17]. The last isoform is the endothelial NOS (eNOS; NOS 3), since it is mostly located in the endothelial cells. The expression of nNOS and eNOS is mainly regulated by Ca^2+^ and calmodulin, which set them apart from iNOS [18], which is activated in the presence of microbial or immunological stimuli [16]. In addition, the NO production by eNOS and nNOS is continuous, but in case of eNOS, it may be enhanced in specific conditions independently of Ca^2+^ signalization. For example, shear stress in the vasculature leads to activation of the phosphoinositide 3-kinase (PI3K) and protein kinase B (Akt) pathways resulting in phosphorylation and activation of eNOS [15]. All three NOS isoforms have been described in the sperm cells of several mammalian species (Table 1) [19]. Interestingly, the pattern of NOS distribution in sperm cells seems to differ across species; for instance, eNOS is localized in the flagellum of human [20], but not bull [21], spermatozoa. Moreover, eNOS is also localized in the equatorial and post-acrosomal regions of morphologically normal human spermatozoa [22]. Aberrant eNOS distribution is often observed in morphologically abnormal spermatozoa and negatively correlates with sperm motility [22]. Furthermore, it is still unclear whether the physiological state (e.g., capacitation) of sperm cells may affect NOS distribution. In a recent study in capacitated boar spermatozoa, Staicu et al. [23] found that the eNOS and nNOS are mainly distributed in the sperm head, whereas iNOS is localized in both the sperm head and the flagellum. The study also suggested a link between NOSs distribution and sperm normal function (capacitation, acrosome reaction, tyrosine phosphorylation, and Ca^2+^ flux). In contrast to boar spermatozoa [23], in epididymal tomcat spermatozoa, all three NOS isoforms are localized in the flagellum and in the cytoplasmic droplet [24]. In murine spermatozoa, the expression of iNOS influences the reproductive outcome [25]. In particular, Yang et al. [25] found that iNOS knockout mice displayed higher fertilization rates, suggesting an iNOS inhibitory effect on sperm fusion with the oocyte. Interestingly, the rate of blastocyst formation was not influenced in any knockout mice. Similarly, the function of pre-ejaculated sperm was unaffected in any NOS knockout [25]. 

### 1.2. H_2_S Metabolism in Spermatozoa

The cellular enzymatic production of H_2_S is mainly ensured by cystathionine β-synthase (CBS), cystathionine γ-lyase (CSE), and 3-mercaptopyruvate sulfurtransferase (3-MST). Both CBS and CSE are pyridoxal 5′-phosphate-dependent enzymes located in the cytosol, while 3-MST is a zinc-dependent enzyme that is mostly found in the mitochondria [29]. Under stress conditions, CSE can be translocated from the cytosol into the mitochondria, producing H_2_S and increasing adenosine triphosphate (ATP) production [30]. Common substrates for H_2_S production are L-homocysteine and L-cysteine, which can be obtained by the methionine transulfuration pathway or directly from the diet [31]. The metabolism of α-ketoglutarate (α-KG) represents an alternative source of H_2_S [32]. The production of H_2_S by 3-MST involves two pathways: a traditional one coupled with cysteine aminotransferase (CAT) and α-KG, and the other one coupled with D-amino acid oxidase (DAO) and D-cysteine [32]. Another pathway for enzymatic production of H_2_S may be the reduction of thiols by catalase [33]. Moreover, H_2_S can be also oxidized by catalase, so this enzyme seems to play an important role in H_2_S metabolism [34]. In addition, mitochondrial complex I is another potentially important source of H_2_S due to the high cysteine concentration compared to the one found in the cytosol. Non-enzymatic synthesis arises from persulfides and polysulfides or from the cellular reservoir of bound sulfur and acid-labile sulfur [29]. In regard to bound sulfur, alkaline conditions (pH > 8.4) within neuronal cells promote the release of H_2_S in the presence of glutathione (GSH) and cysteine [35]. On the other hand, acid-labile sulfides are not a likely source of H_2_S, since their release requires a drop of the pH value to below 5.5 [36]. The catabolism of H_2_S is poorly understood [37] and seems to occur mostly within the mitochondria, thanks to enzymes capable of H_2_S oxidation: sulfide quinone oxidoreductase (SQR), thiosulfate transferase (TST), and sulfite oxidase [32]. Other enzymes also participate in H_2_S catabolism, such as ethylmalonic encephalopathy 1 (ETHE1) protein, which continues the oxidation of sulfide initiated by SQR [38]. Moreover, the enzyme cysteine dioxygenase should be mentioned, as it controls the cellular levels of cysteine, and thus contributes to maintaining low levels of H_2_S/sulfane sulfur pools [38]. The non-enzymatic catabolism pathway occurs via interaction of H_2_S with O_2_, hydrogen peroxide (H_2_O_2_), superoxide (O_2_^−^), and peroxynitrite (ONOO^−^) [32].

There is lack of information regarding the expression and distribution of H_2_S-generating enzymes in sperm cells. To the best of the authors’ knowledge, only one study has quantified the expression of CBS and CSE in sperm samples [39]. In this study, the authors found that oligoasthenozoospermic and asthenospermic men show reduced levels of H_2_S in the seminal plasma compared to fertile men. Interestingly, asthenospermic men show reduced expression of CBS but not CSE. The localization of the H_2_S-generating enzymes within the sperm cells is also still unknown.

## 2. Mechanisms of Action of NO in Spermatozoa

Substantial information is available regarding the role of NO in crucial sperm processes prior to fertilization, such as capacitation, hyperactivation, acrosome reaction, and zona pellucida binding [19,40,41,42]. Furthermore, the role of NO has been widely investigated during semen handling and storage [43,44]. So far, three main pathways of NO within the sperm cell have been established [19].

The primary target of NO is the soluble guanylyl cyclase (sGC) that serves as the NO receptor. The most common sGC isoform found in cytosolic fractions consists of two subunits: α1 and β1. Each subunit contains four domains: N-terminal heme-NO/O_2_ binding (H-NOX), Per/Arnt/Sim domain (PAS), coiled-coil domain (CC; helical d.), and C-terminal catalytic domain [45]. The H-NOX domain of the β1 subunit is the one responsible for the interaction with NO through bounded heme. Upon the binding of NO to the heme group, a cascade of conformational changes of the other domains results in the activation of catalytic activity of the sGC, as demonstrated in vivo using human neuroblastoma-derived cells [46]. The kinetics of the sGC molecule and the interaction with NO was extensively studied by Sürmeli et al. [47] with in vivo implications. The study revealed the relationship between ATP, guanosine-5’-triphosphate (GTP), and NO to the activity of sGC. The ATP binding to the allosteric site (pseudosymmetric to the catalytic domain) gives selectivity of sGC for GTP and affects the enzyme activity at different concentrations of NO [47]. The binding of NO to sGC leads to the production of cyclic guanosine monophosphate (cGMP) [48], which participates in the acrosome reaction of bovine [49] and human [50] spermatozoa. Among cGMP targets, the cyclic nucleotide gated (CNG) channels are one point of interest, since they can be found in the flagellum and affect the Ca^2+^ influx during capacitation of bovine and murine spermatozoa [51,52]. The cGMP also activates cGMP-dependent protein kinase (PKG), an enzyme responsible for phosphorylation of serine/threonine in proteins important for sperm capacitation [19]. Moreover, PKG contributes to the activation of other macroscopic ion currents responsible for maintaining elevated Ca^2+^ levels for longer periods of time during capacitation [52]. An increased production of cGMP also prevents the degradation of cAMP by the phosphodiesterase type 3 (PDE3), as both nucleotides compete for the catalytic site of the enzyme [19]. On the other hand, the intracellular increase of Ca^2+^ may be explained by an extracellular signalization (e.g., progesterone), resulting in sperm-specific Ca^2+^ channel (CatSper) activation and a consequential increase in cGMP production [53].

In addition to the indirect involvement of NO in the cAMP/protein kinase A (PKA) pathway, NO directly acts on adenylyl cyclase (AC) with a dual effect: An activator at small concentrations (murine and human spermatozoa) [54], and an inhibitor at high concentrations (in vitro) [55]. The latter study [55] was performed on transmembrane adenylate cyclase (tmAC), whose function in sperm biology is controversial, despite the fact that all isoforms of tmAC were localized within the cell [56]. In continuation, the tyrosine phosphorylation of proteins is also achieved by the activity of NO on the extracellular signal-regulated kinase (ERK) pathway. NO interacts with the cysteine of Ras proteins, and consequentially several kinases are activated (Raf, MEK, and ERK 1/2) resulting in tyrosine phosphorylation, which contributes to mammalian sperm capacitation [57].

A third mechanism of action occurs at high concentrations of NO, which directly provokes a post-translational modification of proteins, reversibly by S-nitrosylation or irreversibly by tyrosine nitration [40]. Within the human spermatozoa, more than 200 proteins have been identified that are modified by NO via the process of S-nitrosylation [58], which is the covalent union of NO and sulfur of cysteine, forming a nitrosothiol group (-SNO) within the molecule. Moreover, S-nitrosylation is involved in a variety of cellular processes such as energy production, motility, ion channel function, or antioxidative mechanisms [41]. On the other hand, tyrosine nitration is achieved through interaction between NO and ONOO^−^. Interestingly, the levels of tyrosine nitration and the production of ONOO^−^ are increased during mammalian sperm capacitation [41].

In mammals, the major source of NO catabolism seems to be the reaction with O_2_, forming nitrites [29], or with hemoglobin, forming nitrates [59]. The rapid reaction of NO with thiols [29] and other reactive oxygen species (ROS) represents other possible ways of catabolism [17].

## 3. Mechanisms of Action of H_2_S in Spermatozoa

Regarding the targets of H_2_S within the sperm cell, little information is available. Recently, Wang et al. [39] investigated the influence of H_2_S on spermatogenetic failure induced by administration of LPS, which lead to the phosphorylation of mitogen-activated protein kinases (MAPKs), a complex of three downstream enzymes (ERK, C-Jun N-terminal kinase, (JNK), and p38) with pro-inflammatory activity. The injection of the synthetic H_2_S donor GYY4137 attenuated the effect of LPS by modulating the MAPK pathway and affecting the activity of JNK, ERK, and p38 enzymes. Furthermore, the application of the H_2_S donor GYY4137 led to sperm motility improvement in asthenozoospermic men with H_2_S deficiency [39]. In boar and mouse semen, Zhao et al. [60] found that Na_2_S, a fast H_2_S releasing donor, decreases sperm motility by disrupting multiple signaling pathways, which mainly include: decreased ATPase activity, inhibition of Akt, and activation of the adenosine 5‘-monophosphate-activated protein kinase (AMPK) and phosphatase and tensin homologue (PTEN) pathways. The AMPK pathway affects spermatogenesis and performs a crucial role in sperm metabolism and the motility of various mammalian species (e.g., rats, stallions, humans) [61]. On the other hand, the activation of the PI3K/Akt pathway can help to counteract the effects of oxidative stress. Xia et al. [62] observed the activation of the PI3K/Akt pathway in varicocelized (VC) mice after administration of the H_2_S donor (GYY4137) compared to the VC group. The phosphorylation of PI3K p85 and Akt positively correlated with sperm motility, decreased oxidative stress, and reduced epididymal cell apoptosis [62].

However, more potential targets for H_2_S may be expected. H_2_S is known to interact with proteins during post-translational modification [63]. The interaction of H_2_S with cysteine results in the conversion of cysteine -SH groups to -SSH, and the term S-sulfhydration is used to describe this kind of protein modification [64]. Moreover, Mustafa et al. [64], upon the observation of H_2_S interaction with glyceraldehyde 3-phosphate dehydrogenase (GAPDH), suggested H_2_S to be antagonistic to NO, since H_2_S tends to increase cysteine reactivity rather than decrease it, as in the case of NO. This finding may have interesting implications in sperm biology, since GAPDH is a glycolytic enzyme involved in sperm motility [65,66]. In addition, a sperm-specific isoform (GAPDS) with constitutional differences and more specific function is found within sperm cells [67]. The GAPDS is expressed only in male germ cells and performs a narrower range of tasks compared to the somatic isoform (GAPDH). Doubtlessly, the main task is to ensure energy for sperm motion. As a result, the knockout of the gene encoding GAPDS results in a significant motility decrease, while the O_2_ consumption by mitochondria and the ATP production by oxidative phosphorylation (OXPHOS) are maintained [68].

Recently, the term S-sulfhydration has been substituted by a more accurate one, namely persulfidation, as no hydration occurs during the reaction of H_2_S and cysteine -SH group [69,70]. This raises more questions about the direct involvement of H_2_S in cellular signaling, as the sulfur atoms of cysteine and H_2_S are reduced to -2 oxidation state and oxidation to S^-^ is required before persulfidation can occur [70,71]. The slow rate of H_2_S autooxidation, the lower reactivity of H_2_S compared to persulfides/polysulfides, and the low specificity imply that oxidized products of H_2_S (i.e., polysulfides and persulfides) are the actual cellular messengers [70,72]. Mishanina et al. [72] proposed that enzymes producing persulfides, such as sulfurtransferases (e.g., 3-MST, rhodanese), CSE, CBS or SQR, transfer persulfides to another protein directly or via a secondary carrier, which would create targeting specificity. Thus, the persulfide transfer (transpersulfidation) would be the most likely mechanism of signalization of H_2_S.

## 4. The Role of NO and H_2_S in Oxidative Stress

The presence of NO and H_2_S within semen may be linked to physiological processes or pathological states depending on the concentration (Table 2). Whereas at low concentrations ROS play a key role in sperm function (e.g., capacitation, acrosome reaction), above physiological levels they provoke oxidative stress and sperm damage [73,74]. Apart from ROS, reactive nitrogen species (RNS) [75] and reactive sulfur species (RSS) [76] are also involved in several cellular processes. To maintain the balance between physiological signal transduction and over-accumulation of reactive species, antioxidants such as super oxide dismutase (SOD), catalase, or the glutathione peroxidase (GPX)/glutathione reductase (GR) system are present within the seminal plasma [77]. Moreover, the sperm cell itself has an intrinsic antioxidant system, involving antioxidants such as peroxiredoxins and thioredoxins, in addition to the above-mentioned seminal plasma antioxidants [78]. Nevertheless, it should be emphasized that sperm cells possess limited antioxidant capacity due to the low content of cytoplasm and the high content of polyunsaturated fatty acids (PUFA), which make the male gamete vulnerable to oxidative stress [73]. A study by Moretti et al. [79] demonstrates that the increased ROS production in infertile men leads to impairment of sperm parameters (e.g., motility and viability) and alteration of the antioxidant system within the cell. As mitochondria are the main source of ROS within the spermatozoon, as well as a major source of energy for movement, the decrease in sperm motility in response to oxidative stress may be linked to alterations of mitochondrial activity [80].

### 4.1. NO and Reactive Nitrogen Species

NO is a free radical representing the main source of RNS, which originate from the interaction of NO with O_2_ and O_2_^−^ to produce nitrogen dioxide (NO_2_), dinitrogen trioxide (N_2_O_3_), dinitrogen tetraoxide (N_2_O_4_), ONOO^−^, and nitroxyl (HNO) [40]. Ultimately, excessive RNS can be responsible for lipid, protein, and DNA impairment [81]. NO is the least reactive radical often connected with PUFA peroxidation. As a free radical, increased concentrations of NO within the sperm cell are associated with male infertility [79]. In this way, aminoguanidine, an NOS inhibitor, protects the sperm cells against the detrimental consequences of oxidative stress both in vivo and in vitro [82,83]. Yet, it should be mentioned that NO may also stop radical chain propagation through interaction with the lipid peroxyl radical (an intermediate of lipid peroxidation) to form oxidized forms of nitrosated fatty acid species [84]. Apart from its physiological role, NO pathological accumulation at µM concentrations in mitochondria inhibits cellular respiration, while at mM concentrations it may also lead to membrane hyperpolarization, cytochrome c release, and apoptosis [10]. The inhibition of mitochondrial respiratory activity by NO itself is done through reversible inhibition of complex IV upon the binding of NO to the heme group of cytochrome oxidase [84]. In addition to NO, the free radical ONOO^−^ is one of the most potent RNS involved in various signaling pathways, and has potential pathological effects when left uncontrolled by the antioxidant cellular defense [85]. The overproduction of ONOO^−^ leads to the inhibition of mitochondrial activity through the inactivation of electron transport complexes I (NADH dehydrogenase) and II (succinate dehydrogenase). The function of SOD can be also affected by ONOO^−^ through tyrosine nitration [84]. Various types of SOD are known, of which two types are found in eukaryotic organisms: Mn-SOD located in the mitochondria and Cu/Zn-SOD mostly located in the cytosol [86]. Mn-SOD is inactivated by ONOO^−^ [84]. The influence of ONOO^−^ overproduction on human spermatozoa was investigated by Uribe et al. [87], revealing a decrease in the mitochondrial membrane potential and motility. These observations led to the hypothesis that decreased ATP production could be behind the observed effects. This hypothesis was later confirmed by the same research group [88], as the application of peroxynitrite interfered with ATP production via OXPHOS, and also via glycolysis. Moreover, thiol oxidation, resulting from the reaction of ONOO^−^ with sulfhydryl groups of cysteine, was related to decreased sperm motility. The process affected both the sperm head and the principal piece, and as a possible explication of motility loss, a thiol oxidation of the sperm axoneme was suggested [89]. In addition, Uribe et al. [90] observed mitochondrial permeability transition (MPT) under nitrosative stress with biochemical traits of MPT-driven necrosis. On the contrary, Serrano et al. [91] found that, although peroxinitrite induces oxidative stress in boar sperm, leading to lipid peroxidation and motility loss, it does not affect mitochondrial membrane potential.

### 4.2. H_2_S and Reactive Sulfur Species

The most recent and complex definition describes the RSS as those molecules which contain at least one redox-active sulfur atom or sulfur-containing functional group in their structure, and are capable of either oxidizing or reducing biomolecules under physiological conditions to trigger or propagate a noticeable cellular signal or wider biological event [92]. The need for this new definition comes from the extensive research done in the area of cellular signaling involving RSS. Mishanina et al. [72] list a wide range of biologically active RSS with H_2_S as a common precursor. Like RNS and ROS, the concentration of RSS is crucial for physiological activity, as in supraphysiological concentrations, RSS exert a negative effect on sperm cells [40]. In a study by Wang et al. [39], asthenozoospermic men showed decreased H_2_S concentrations in seminal plasma, and the application of a H_2_S donor (GYY4137) improved the total and progressive sperm motility. In the same study, the negative effect on human sperm motility and hypermotility was seen after 5 µM NaHS treatment, which probably caused the fast release of H_2_S in a supraphysiological concentration. Similarly, Zhao et al. [60] reported that the administration of Na_2_S, both in vitro (25–100 µM) and in vivo (10 mg/kg of body weight), led to negative effect on boar and mouse sperm motility, respectively. The observed negative effects of H_2_S donors may be due to the inhibition of ATP production. Particularly, the inhibition of mitochondrial complex IV takes place when using an NaHS donor in concentrations exceeding 10 µM in various cell lines [93]. Finally, high concentrations of a H_2_S donor (50 µM Na_2_S) promote oxidative stress, measured as the concentration of H_2_O_2_, in boar sperm samples [60].

### 4.3. H_2_S Antioxidant Properties

Great focus has been dedicated to the antioxidant properties of H_2_S as a reducing agent (Table 2) [94]. At low concentrations H_2_S and its dissociated form, (HS^-^), can directly scavenge ROS and RNS (e.g., O_2_^−^, H_2_O_2_ or peroxynitrates) [35]. Bearing in mind the very low H_2_S cellular concentration (sub-micromolar), the direct scavenging activity seems to be of lesser importance compared to other antioxidants (e.g., GSH) [35,95]. On the other hand, indirect augmentation of antioxidant capacity has been documented in several studies. In a study by Li et al. [96], the application of NaHS as a H_2_S donor led to increased SOD activity and decreased ROS levels in testicular germ cells exposed to heat stress. Moreover, mitochondrial dysfunction characterized by increased ATP depletion, O_2_ consumption, and ROS generation was also reduced after NaHS application. The results also indicated that H_2_S may prevent cellular apoptosis. In a similar study, Ning et al. [97] used another H_2_S donor, GYY4137, to test its effect on heat-induced damage in testicular cells. In agreement with Li et al. [96], H_2_S donor administration led to increased SOD expression and reduced the number of apoptotic cells. The authors also measured the expression of several proteins of the mitochondrial apoptotic pathway: Bax, Bcl-2, and caspase 3. The application of GYY4137 reduced the expression of Bax in heat-exposed testicular cells and preserved the expression of Bcl-2 compared to the group without treatment [97]. The ratio between Bax (pro-apoptotic factor) and Bcl-2 (anti-apoptotic factor) protein is crucial in apoptosis activation [98]. As a consequence, the authors also found that the expression of caspase 3 was also reduced in the GYY4137-treated group [97]. Caspase 3 is a signaling enzyme of various pathways, whose activation leads to inevitable apoptosis [99]. The effects observed by Ning et al. [97] are attributed to the increased expression of heat shock protein 70 (HSP 70) after GYY4137 application. The expression of HSP 70 helps to prevent cell apoptosis during temperature-induced stress conditions in testicular cells [100], preserves sperm motility in cryopreserved bull spermatozoa [101], and protects proteins and DNA under stress conditions [102]. Using antioxidant sericin, the increased expression of HSP 70 led to improved semen quality after cryopreservation [103].

## 5. NO and H_2_S Interactions

**Figure 1:**
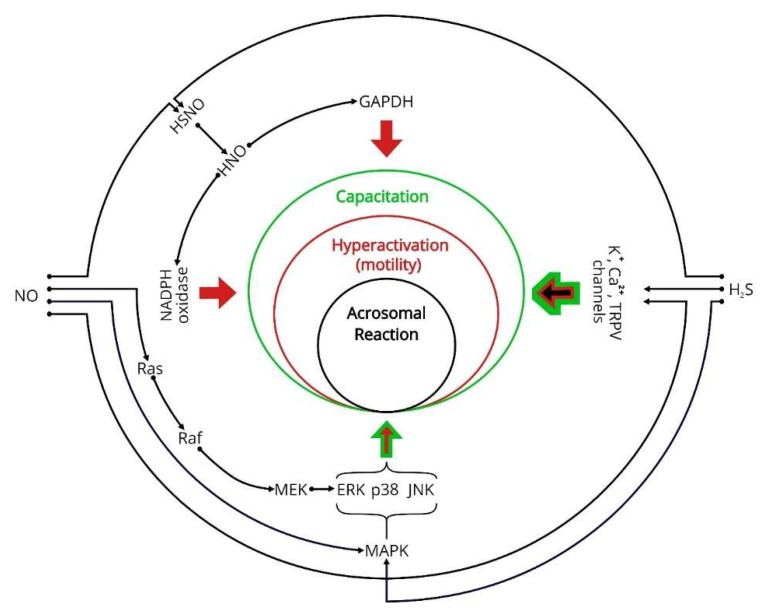
Common targets of nitric oxide (NO) and hydrogen sulfide (H_2_S). The scheme displays the cohesion of H_2_S and NO common targets within a cell, focusing on the most sperm-relevant enzymes and proteins. The function of NADPH oxidase and GAPDH directly affects sperm motility, as the latter requires ATP production. The sperm ion channels affect not only sperm function (capacitation, hyperactivation, acrosomal reaction), but also the outcome of the fertilization process. The MAPK complex influences the capacitation and hyperactivation of sperm cells. Colors of arrows indicate the relation with sperm biological process marked by the corresponding color. ERK, extracellular signal-regulated kinase; GAPDH, 3-phosphate dehydrogenase; HNO, nitroxyl; HSNO, thionitrous acid; H_2_S, hydrogen sulfide; JNK, C-Jun N-terminal kinase; MAPK, mitogen-activated protein kinases; MEK, MAPK/ERK kinase; NADPH, nicotinamide adenine dinucleotide phosphate; NO, nitric oxide; Raf, rapidly accelerated fibrosarcoma kinase; TRPV, transient receptor potential vanilloid.

**Figure 2:**
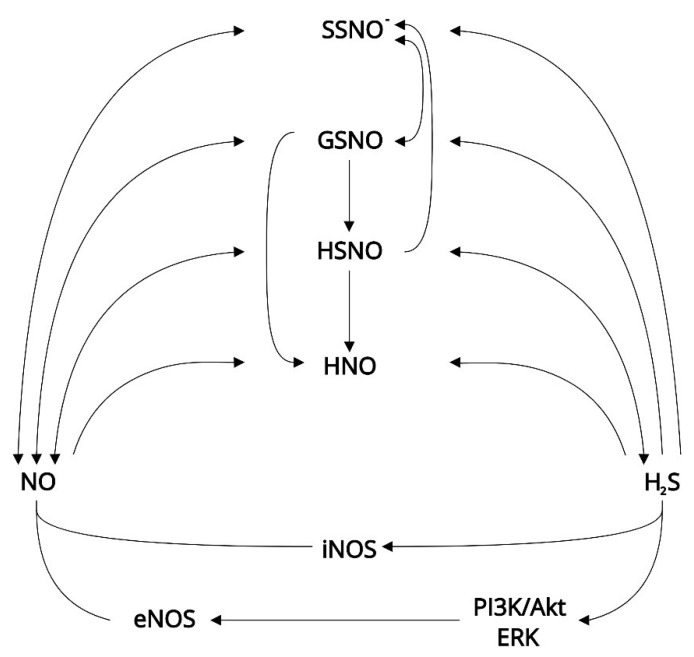
A brief insight into the interactions between NO and H_2_S that might be relevant for sperm biology. Akt, protein kinase B; eNOS, endothelial nitric oxide synthase; ERK, extracellular signal-regulated kinase; GSNO, S-nitrosoglutathione; HNO, nitroxyl; HSNO, thionitrous acid; H_2_S, hydrogen sulfide; iNOS, inducible nitric oxide synthase; NO, nitric oxide; PI3K, phosphoinositide 3-kinase. [32,50,116,117,118,119,123,124,125,126,127,128,129,130].

There is growing evidence indicating that H_2_S and NO share common targets and interact with each other [104]. Most information about the interactions of H_2_S and NO come from research on the cardiovascular system. The studies dedicated to this topic demonstrate the interaction on several levels: shared signaling targets (Figure 1), metabolic regulation of each other, and interaction between metabolites of both gasotransmitters (Figure 2) [59]. For example, the interaction of H_2_S and NO leads to the formation of polysulfides, which are more reactive than H_2_S, and thus seem to be novel RSS signal conductors [70].

With respect to the common signaling targets for H_2_S and NO, the MAPK pathway is one point of interest. The MAPK pathway includes four main cascades, namely, ERK 1/2, JNK, p38, and ERK 5, and it is known to participate in sperm capacitation, motility, and acrosome reaction [105]. While H_2_S decreases phosphorylation by MAPK in the testis [39], NO activates MAPK participating in the tight-junction dynamics of Sertoli cells [106]. This MAPK regulation by H_2_S and NO may also be of interest regarding sperm cells, as it is a crucial pathway affecting sperm motility, morphology, and capacitation [57,107]. For the first time in human spermatozoa, Silva et al. [107] identified JNK, which represent a subfamily of MAPKs also referred to as stress-activated protein kinases (SAPKs), as they are activated by phosphorylation under stress conditions (e.g., oxidative stress). The same authors observed a negative correlation of JNK phosphorylated levels with total and progressive motility. Furthermore, the application of NaHS in mice decreased the activity of MAPKs in the blood–testis barrier of samples exposed to oxidative stress induced by LPS [39]. Thus, it seems that the phosphorylation of MAPKs is attenuated by H_2_S. On the other hand, exposure of cells to peroxynitrate activates all three of the major subfamilies of MAPKs (p38, JNK, ERK) in rat liver epithelial cells [108]. Yet, the effect of the two gasotransmitters on the MAPK pathway within a sperm cell remains to be investigated. Other common targets in somatic cells for both gasotransmitters are the Ca^2+^ channels [29,35] and K^+^ channels [29,35,59]. The regulation of Ca^2+^ currents in sperm is of particular interest, as CatSper are involved not only in sperm capacitation [109], but also in sperm hyperactivation, acrosome reaction, and chemotaxis [53,110]. The hypothesis of NO involvement in chemotaxis through affection of the ion channel function may seem intriguing, as Miraglia et al. [111] observed a positive influence of NO on sperm migration. On the other hand, a recent study by Wilińsky et al. [112] showed only temporal negative influence of H_2_S on sperm chemotaxis, probably due to motility inhibition. The specific mechanism and extent of involvement of the CatSper channels in the previously mentioned processes is still a matter of debate [113,114]. In addition, the opening of K^+^ channels induces membrane hyperpolarization, representing the predominant process during capacitation. The regulation of K^+^ channels also affects ATP generation by mitochondria, and thus the activation promotes progressive sperm movement, together with hyperactivity [115].

Attention should also be given to the transient receptor potential (TRP) channels, which affect male fertility potential, starting from spermatogenesis, through sperm maturation, to sperm function. The TRP channels are involved in sperm thermotaxis, forming a group of 30 Ca^2+^ ion channels, which can be divided into seven families [116,117]. Some channels of the subfamily of TRP vanilloid (TRPV) can be activated by H_2_S [29] and NO through S-nitrosylation [118]. The ion channel TRPV type 4 (TRPV4) was very recently demonstrated to participate in human sperm capacitation and hyperactivation [119]. The TRPV4 channel function is temperature dependent and is probably modulated by tyrosine phosphorylation [119]. Following the authors’ model, TRPV4 mediates Na^+^ influx and the consequential membrane depolarization necessary for activation of other crucial capacitation-related ion channels (e.g., CatSper). The authors immunolocalized TRPV4 in the flagellum and acrosome of human spermatozoa. Another TRP channel (TRPV1) was immunolocalized by Kumar et al. [120] in the acrosome and in the flagellum of bull spermatozoa. The authors observed a correlation of TRPV1 with progressive sperm motility, hyperactivity, capacitation, and acrosome reaction. TRPV1 was also observed to play an important role in the capacitation of boar spermatozoa [121]. The activation of TRPV1 leads to membrane depolarization through Na^+^ influx and the consequential activation of voltage-gated Ca^2+^ channels. The same effect was also observed in mouse spermatozoa [122]. In a study by Bernabò et al. [121], the TRPV1 localization displayed two patterns in ejaculated spermatozoa: in the majority of spermatozoa, TRPV1 was found in the post-acrosomal region, while around 20% of spermatozoa had TRPV1 distributed over the acrosome and in the proximal segment of the midpiece. The authors observed a dramatic shift of this distribution pattern after capacitation, describing the relocation of TRPV1 to the acrosome and midpiece. Yet the regulation of the TRPV channel by H_2_S and NO in spermatozoa of different species remains to be investigated.

A regulatory effect of H_2_S on NO production may result from the ability of H_2_S to activate the PI3K/Akt and ERK pathways [60]. Using various H_2_S donors in CSE knockout mice, H_2_S activates eNOS in myocardial cells [123]. The enzymes ERK 1/2 were reported to enhance eNOS sensitivity to Ca^2+^ stimulation in the endothelial cells of the uterine artery [124]. The release of NO upon the activation of MEK/ERK1/2 and PI3K/Akt-dependent eNOS serine 1179 phosphorylation was also observed after H_2_O_2_ application [125], which describes a cellular mechanism of adaptation to oxidative stress. In contrast, the application of the H_2_S donors NaHS and diallyl trisulfide leads to the inhibition of iNOS during inflammation [126]. However, the effect of the interaction between NOS and H_2_S is still unclear, indifferent of cell type [127].

A direct interaction between H_2_S and NO radicals and their metabolites (e.g., nitrate, nitrite, peroxinitrates) results in the formation of potentially important signaling molecules such as nitrosothiols, thionitrous acid (HSNO), or nitroxyl (HNO) [59,128]. The interaction between NO and H_2_S is currently being intensively investigated, as it represents a very complex topic of great physiological importance and results in a plethora of possible outcomes [129]. For instance, HS^−^ reacts with ONOO^−^, forming HSNO [59], which seems to be another important source of NO and HNO [130]. In addition, the reaction of HS^-^ with S-nitrosothiol (SNO) and S-nitrosoglutathione (GSNO) generates several other metabolites (e.g., sulfinyl nitrite (HSNO_2_) and HSNO) [127]. Within the cardiovascular system, the role of HNO in cellular physiology has received considerable attention [59], with possible interesting implications for sperm cells. Using a HNO donor (Angeli’s salt), Andrews et al. [131] demonstrated for the first time that it acts through the sGC/cGMP pathway. HNO also protects PUFA from peroxidation due to its antioxidant properties [132]. The protective ability of HNO should also be considered in the case of the sperm plasma membrane, as it contains a high amount of PUFA [73]. On the other hand, HNO can increase intracellular levels of H_2_O_2_ by inhibiting its degradation, and it also reacts with thiol proteins, such as GAPDH, decreasing its activity [132,133]. Sperm-specific GAPDH (GAPDS) is particularly important in sperm cell energetic metabolism [68]. It has been proposed that interaction of NO with H_2_S may result in GSNO formation [37]. Yet, the reaction of nitrous acid (HNO_2_) with GSH seems to be the most relevant in physiological conditions, compared to the reaction of GSH and NO, which represents another alternative for in vivo GSNO formation [134]. It seems that GSNO serves as an intracellular storage for NO, which can be released by the reaction with GPX or thioredoxin reductase [59]. GSNO can also release stored NO upon reaction with H_2_S or HS^-^ [135], and can also lead to formation of polysulfane species [136]. In addition, Berenyiova et al. [137] proposed that sulfide reaction with GSNO may lead to HNO synthesis. Although HNO was observed to inhibit nicotinamide adenine dinucleotide phosphate (NADPH) oxidase (Nox 2) in the vascular system [59], the form and role of NADPH oxidase in spermatozoa is unclear [11]. Only the isoform Nox 5 has been found in the testis [11] and in human spermatozoa, where it was localized in the flagellum, midpiece, and acrosome and was positively associated with motility [138]. Recently, nitrosopersulfide (SSNO^−^) was suggested as a more probable, effective, resistant, and specific NO donor than GSNO [139,140]. It was also suggested that SSNO^-^ is formed in the presence of excessive sulfide, in addition to the other ways of formation [139]. On the other hand, Wedmann et al. [141] proposed that under in vivo physiological conditions, HSNO/SNO^−^ is the most probable signaling molecule (via trans-nitrosation), which can also cause HNO formation.

## 6. Conclusions

In conclusion, the roles of H_2_S and NO in sperm cells still leave many unanswered questions. Surprisingly, even after two decades of intensive investigation, the exact mechanism of action of H_2_S is still unclear. The delicately tuned relationship and wide range of molecular targets of these two gasotransmitters within the cell highlight the necessity for further research. Growing evidence indicates that the research on the male gamete should not only take into account the sole action of each gasotransmitter, but it should also focus on investigating the interaction between NO and H_2_S.

## Figures and Tables

**Table 1:** The presence and localization of nitric oxide synthases (NOSs) in sperm of different species.

Species	NOS Isoform	Localization	Reference
Man	nNOS	Head, tail	[26]
	eNOS	Head	[22]
Mouse	nNOS, iNOS, eNOS	n/a	[27]
Bull	nNOS	Head, tail	[21]
	eNOS	Head	
Boar	nNOS	Head	[23]
	iNOS	Head, tail	
	eNOS	Head	
Stallion	nNOS, eNOS	n/a	[28]
Tomcat	nNOS, iNOS, eNOS	Tail, cytoplasmic droplet	[24]

This table was adapted from Staicu and Matas Parra [19] and modified for the purpose of this review. n/a, not available; NOS, nitric oxide synthase; nNOS, neuronal NOS; iNOS, inducible NOS; eNOS, endothelial NOS.

**Table 2:** The effects of nitric oxide (NO) and hydrogen sulfide (H_2_S) on cellular function.

PHYSIOLOGICAL CONCENTRATION		SUPRAPHYSIOLOGICAL CONCENTRATION	
NO	H_2_S	NO	H_2_S
↓ lipid peroxidation*	ROS scavenging activity* ↑ **antioxidant capacity** ○ ↑SOD activity ↑ **mitochondrial activity** ↑ **sperm motility** ↑ **DNA integrity** **apoptosis prevention** ○ ↑ HSP 70 expression ○ ↓ Caspase 3 expression ○ Bax/Bcl-2 ratio preservation **Cryoprotection** ○ ↑ HSP 70 expression ▪ ↑ sperm motility ▪ ↑ membrane integrity ▪ ↑ DNA integrity ▪ ↓ % abnormal sperm	↑ **lipid peroxidation** ↑ **DNA damage** ↑ **protein damage** ↑ apoptosis* ○ membrane hyperpolarization* ○ cytochrome C release* ↓ **mitochondrial activity** ○ complex IV inhibition* ↑ **ONOO^-^ generation** ○ mitochondrial activity inhibition ▪ complexes I and II inhibition* ▪ Mn-SOD inactivation* ▪ Succinate dehydrogenase inactivation* ○ ↓ glycolysis ○ ↑ thiol oxidation	↓ **sperm motility** ↑ **ROS levels** ↓ **mitochondrial activity** ○ Complex IV inhibition*

* Effects seen in other systems rather than just the male reproductive system. Bax, Bcl-2-associated X protein; Bcl-2, B-cell lymphoma 2 protein; HSP, heat-shock protein; ROS, reactive oxygen species; SOD, superoxide dismutase; ONOO^−^, peroxynitrite. While bold letter indicates topic within the table, circles and squares indicates 1st and 2nd level subtopics.

## Abbreviations

3-MST	3-mercaptopyruvate sulfurtransferase
AC	adenylyl cyclase
Akt	protein kinase B
AMPK	adenosine 5‘-monophosphate–activated protein
ATP	adenosine triphosphate
Bax	Bcl-2-associated X protein
Bcl-2	B-cell lymphoma 2 protein
CAT	cysteine aminotransferase
CatSper	sperm specific Ca^2+^ channels
CBS	cystathionine β-synthase
CC	coiled-coil domain
cGMP	cyclic guanosine monophosphate
CNG	cyclic nucleotide gated (channels)
CSE	cystathionine γ-lyase
DAO	D-amino acid oxidase
eNOS	endothelial nitric oxide synthase
ERK	extracellular signal-regulated kinase
ETHE1	ethylmalonic encephalopathy 1 protein
GAPDH	glyceraldehyde 3-phosphate dehydrogenase
GAPDS	sperm-specific glyceraldehyde 3-phosphate dehydrogenase
GPX	glutathione peroxidase
GR	glutathione reductase
GSH	glutathione
GTP	guanosine-5’-triphosphate
H-NOX	N-terminal heme-NO/O_2_ binding (domain)
HSP	heat shock protein
iNOS	inducible nitric oxide synthase
JNK	C-Jun N-terminal kinase
MAPK	mitogen-activated protein kinases
MEK	MAPK/ERK kinase
MPT	mitochondrial permeability transition
NADH	nicotinamide adenine dinucleotide
NADPH	nicotinamide adenine dinucleotide phosphate
nNOS	neuronal nitric oxide synthase
NOS	nitric oxide synthase
Nox_2	nicotinamide adenine dinucleotide phosphate oxidase 2
OXPHOS	oxidative phosphorylation
PAS	Per/Arnt/Sim (domain)
PDE3	phosphodiesterase type 3
PI3K	phosphoinositide 3-kinase
PKG	cGMP-dependent protein kinase
PTEN	phosphatase and tensin homologue
PUFA	polyunsaturated fatty acids
Raf	rapidly accelerated fibrosarcoma kinase
RNS	reactive nitrogen species
ROS	reactive oxygen species
RSS	reactive sulfur species
SAPK	stress-activated protein kinases
sGC	soluble guanylyl cyclase
SQR	sulfide quinone oxidoreductase
TRP	transient receptor potential (channels)
TRPV	TRP vanilloid (channels)
TST	thiosulfate transferase
VC	varicocelized
α-KG	α-ketoglutarate
